# PepMapper: A Collaborative Web Tool for Mapping Epitopes from Affinity-Selected Peptides

**DOI:** 10.1371/journal.pone.0037869

**Published:** 2012-05-25

**Authors:** Wenhan Chen, William W. Guo, Yanxin Huang, Zhiqiang Ma

**Affiliations:** 1 School of Computer Science and Information Technology, Northeast Normal University, Changchun, China; 2 School of Information & Communication Technology, Central Queensland University, Rockhampton, Queensland, Australia; 3 National Engineering Laboratory for Druggable Gene and Protein Screening, Northeast Normal University, Changchun, China; 4 Key Laboratory of Intelligent Information Processing of Jilin Universities, Northeast Normal University, Changchun, China; Technical University of Denmark, Denmark

## Abstract

Epitope mapping from affinity-selected peptides has become popular in epitope prediction, and correspondingly many Web-based tools have been developed in recent years. However, the performance of these tools varies in different circumstances. To address this problem, we employed an ensemble approach to incorporate two popular Web tools, MimoPro and Pep-3D-Search, together for taking advantages offered by both methods so as to give users more options for their specific purposes of epitope-peptide mapping. The combined operation of Union finds as many associated peptides as possible from both methods, which increases sensitivity in finding potential epitopic regions on a given antigen surface. The combined operation of Intersection achieves to some extent the mutual verification by the two methods and hence increases the likelihood of locating the genuine epitopic region on a given antigen in relation to the interacting peptides. The Consistency between Intersection and Union is an indirect sufficient condition to assess the likelihood of successful peptide-epitope mapping. On average from 27 tests, the combined operations of PepMapper outperformed either MimoPro or Pep-3D-Search alone. Therefore, PepMapper is another multipurpose mapping tool for epitope prediction from affinity-selected peptides. The Web server can be freely accessed at: http://informatics.nenu.edu.cn/PepMapper/

## Introduction

Epitope mapping from affinity-selected peptides has been proven to be a useful approach in identifying native epitopes for immunological applications in recent years [1], [2], [3], [4], [5], [6], [7], [8]. Affinity-selected peptides which are derived from phage-display experiments, also known as mimotopes, are assumed to have similar components with the native epitope [9]. Various ways have been proposed to map the mimotopes back to the genuine epitope. These methods were reviewed and compared in some recent literature [10], [11]. In general, they can be categorized as sequence based [12], motif based [7], [13], physicochemical properties based [14], and graph search based [4], [15], [16] methods. Graph search methods are among the most efficient ways in epitope mapping demonstrated in many recent publications [4], [15], [16] because they take advantages of more information provided by using both the 3D structure of a protein than using the traditional amino acid sequence and the information from mimotope set.

The essential idea of graph search methods is to find a group of simple paths on a graph generated from the residues on the surface of a protein and find out some paths from the graph best matched to the query peptides derived from in vitro screening against a target antibody [6]. Searching in Pep-3D-Search [4] is achieved through an algorithm based on ant colony optimization (ACO) whereas PepSurf [6] realizes the mapping using a dynamic programming based stochastic color-coding algorithm. However, finding a simple path on a graph is computationally intractable for any large scale of searching problem. For example, PepSurf takes a few hours to get the result for a peptide of 14 or 15 amino acids.

MimoPro[16] has brought improvement on processing speed and sensitivity over both PepSurf and Pep-3D-Search. It uses an adaptable distance threshold (ADT) regulated by an appropriate compactness factor to define a graph from a small patch on the surface of a protein. Such a regulated graph contains a certain number of edges, which can guarantee that searching through the graph is more efficient. On average, MimoPro achieved the best performance among the three, but individual cases produced mixed outcomes. This indicates that no one dominates over others in all circumstances but each has its advantage in dealing with particular cases.

Perhaps the best strategy is to combine two or more methods together to deal with various cases of epitope-peptide mapping in practice. Pepitope [17] combined both PepSurf and Mapitope [1] together as a Web tool for epitope-peptide mapping so as to complement with each other. The algorithm used in PepSurf maps the affinity-selected peptides directly back to the protein surface. The most significant alignments are then clustered into a patch, from which the epitope location is inferred. In Mapitope, each peptide is first deconvoluted to amino acid pairs, and those pairs of residues that are significantly overrepresented in the panel of peptides are then identified. Epitopic regions are finally predicted through searching for a cluster of those enriched pairs on the 3D structure of the antigen.

Although significant progress has been made in epitope prediction through mimotope mapping, we must acknowledge that the performance of any algorithm devised and any tool developed so far was evaluated based on the outcomes of very limited test cases in which the epitopic region must be known and both the structure of the antigen and the peptide set derived from high-throughput screening must be available. If a single method is applied to a case in which the epitopic region is unknown, the mapping simply returns a candidate epitope (or none) with aligned paths formed by the antigen surface residues (or none). Such candidate epitope will become the focus of further investigation through other means.

If no any single experimentally derived peptide is related to any region on the antigen, it only indicates that this method is not applicable for the case through the mapping. However, it does not mean that no interacting epitope exists on the antigen, which may be detected by other methods. In this regard, the likelihood of finding a genuine epitopic region on an antigen should be higher if more associated peptides can be detected through the mapping. Furthermore, if more mapping methods can be combined together for exploring as many associated peptides as possible through the mapping, the likelihood of finding a genuine epitopic region on the antigen should be enhanced.

On the other hand, users of a mapping tool would prefer to know some sort of certainty about the candidate epitope determined by the associated peptides through the mapping in relation to the likelihood of being a genuine epitope. In other words, some kind of verification on the candidate epitope, if not a confirmation, will be much helpful for the users to make an initial assessment on the quality of the candidate. A single method cannot achieve this goal by self verification, but a combined approach of two or more independent methods would be able to provide mutual verification on the candidate of the same case. Web tools realizing such collaborative concept have not been tried so far.

In this paper, we report our effort on combining both MimoPro and a modified version of Pep-3D-Search together to realize such a collaborative Web tool for supporting users in peptide-epitope mapping. In addition to the process of either MimoPro or Pep-3D-Search alone, the combined operation of *Union* captures the concept of exploring as many associated peptides as possible from both methods. The concept of mutual verification is realized by the combined operation of *Intersection* from both methods.

In the next section, we introduce the processes of MimoPro, Pep-3D-Search, and the combined approach of PepMapper. Their online implementations are then briefly outlined. Construction of test cases and assessment of mapping are incorporated with discussions of the experimental results. Conclusions are finally drawn.

## Methods

### Pep-3D-Search

**Figure 1:**
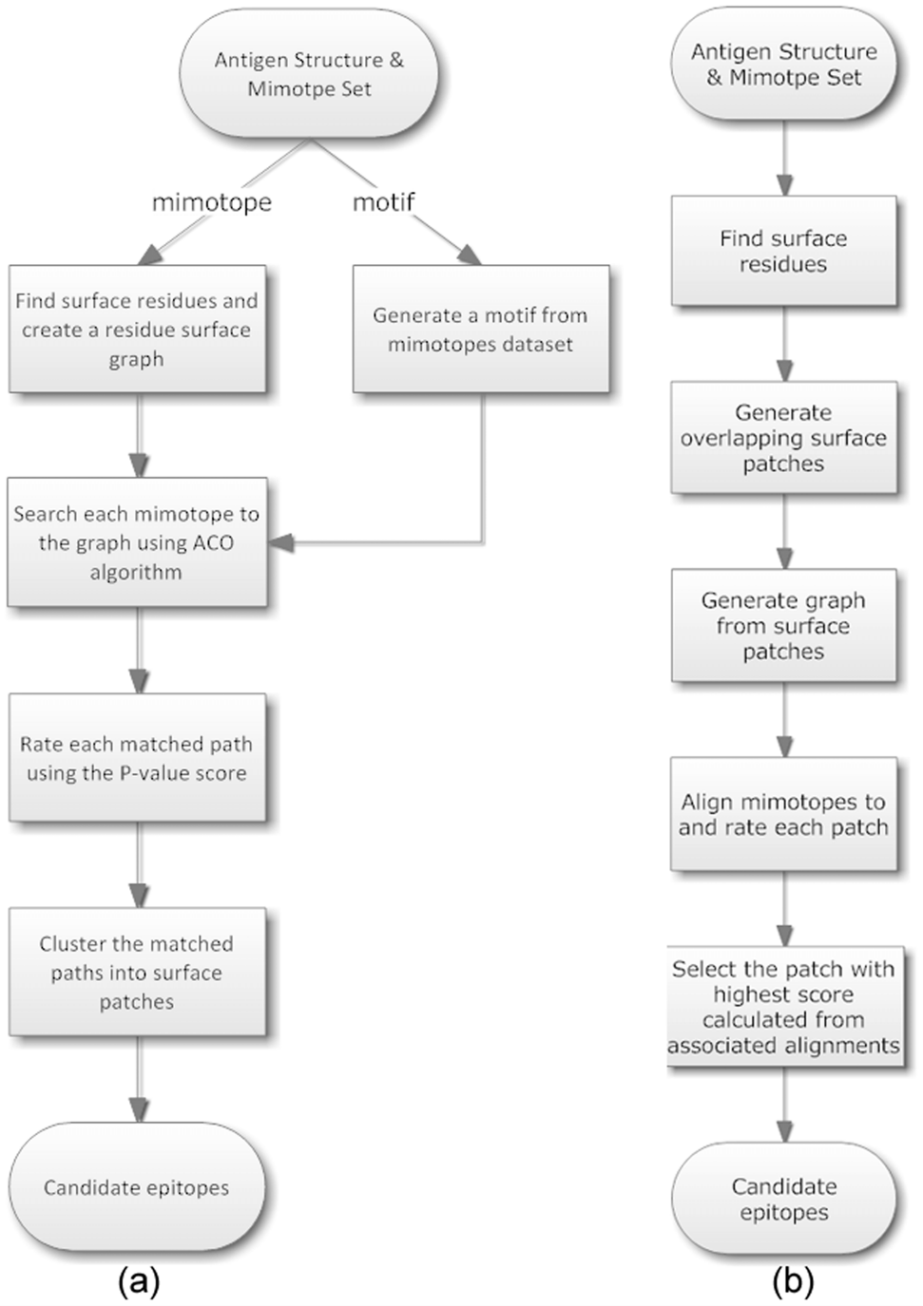
Flow charts of Pep-3D-Search (a) and MimoPro (b).

The process of Pep-3D-Search [4] is illustrated in Figure 1a. Given a 3D structure of an antigen, Pep-3D-Search identifies all the surface residues and creates a surface graph using those residues. An ACO algorithm is then used to search the matched paths on the antigen surface with respect to the query peptides or motif. Each matched path is then rated by its P-value score [4]. A set of highly rated paths are selected to create a weighted graph of resultant paths. The Depth-First Search (DFS) algorithm is finally used to screen and cluster this weighted graph to define the candidate epitopes.

The process of Pep-3D-Search has two unique features. Firstly, Pep-3D-Search is able to deal with both mimotope searching and motif mapping on the residue surface graph. Secondly, the adoption of ACO algorithm allows longer mimotopes or motifs to be processed with reasonable efficiency. The performance of Pep-3D-Search assessed by a few comparative studies [16] seems to be above the average level.

### MimoPro

The process of MimoPro [16] is illustrated in Figure 1b. Initially, the surface of a protein is divided into some overlapping patches and each patch is centered at atom *C_β_* of a surface residue with a radius of 15 Å. This radius allows most epitopes to be encompassed in such a patch [16]. Secondly, each surface patch is further transformed to a graph bounded by neighbor amino acids that are determined using a parameter called adaptive distance threshold (ADT). Afterwards a patch-based complete graph search algorithm is utilized to find the best alignment for each mimotope sequence in each graph. During this iteration, similarity between a path and the corresponding mimotope is rated. Finally the patch with the highest score is selected as a potential candidate for the native epitope.

This approach has some new features different from other similar methods. Firstly, the ADT that is changeable in different regions of a protein is introduced in generating a graph from a surface patch. Such a distance threshold is adjustable so that a longer distance is used in loose regions of an antigen to include more useful connections whereas a shorter distance is adopted in dense regions to preclude some insignificant connections. Secondly, a compactness factor is introduced to make sure that all resultant graphs share a uniform compactness so that searching over any regulated graph is simpler and faster compared with previous methods. Thirdly, the adopted algorithm not only employs dynamic programming (DP) to reduce repeating searches and prune some insignificant paths encountered in the traditional search algorithm, but also introduces the branch and bound method to optimize the candidate set of rated paths during the DP process. The performance of MimoPro assessed by a few comparative studies [16], [18], [19] shows that MimoPro seems to be the most sensitive tool on average among the compared tools.

### PepMapper

**Figure 2:**
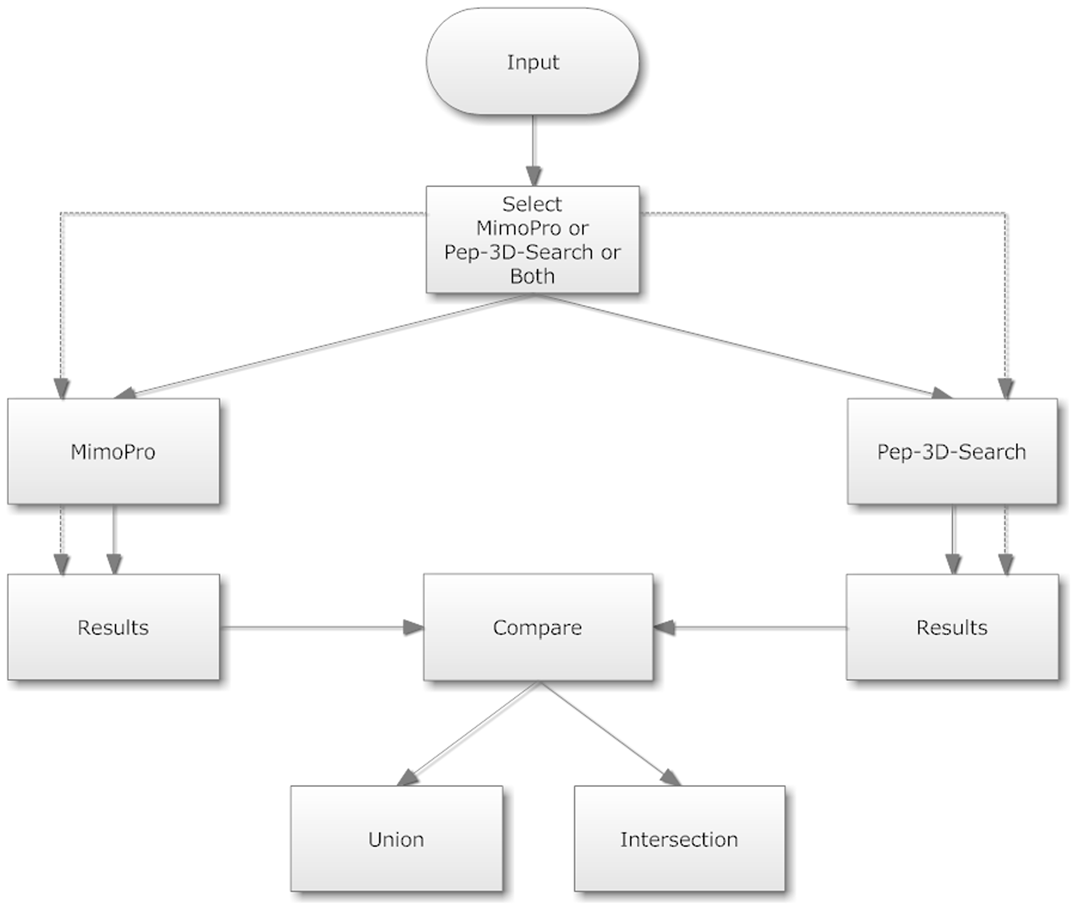
Flow chart of PepMapper. Solid lines indicate the process sequences of PepMapper whereas dashed lines are for that of either MimoPro or Pep-3D-Search alone.

PepMapper provides users with a united platform to conduct peptide-epitope mapping through either MimoPro or Pep-3D-Search or both for different purposes. The processes of PepMapper are illustrated in Figure 2.

**Figure 3:**
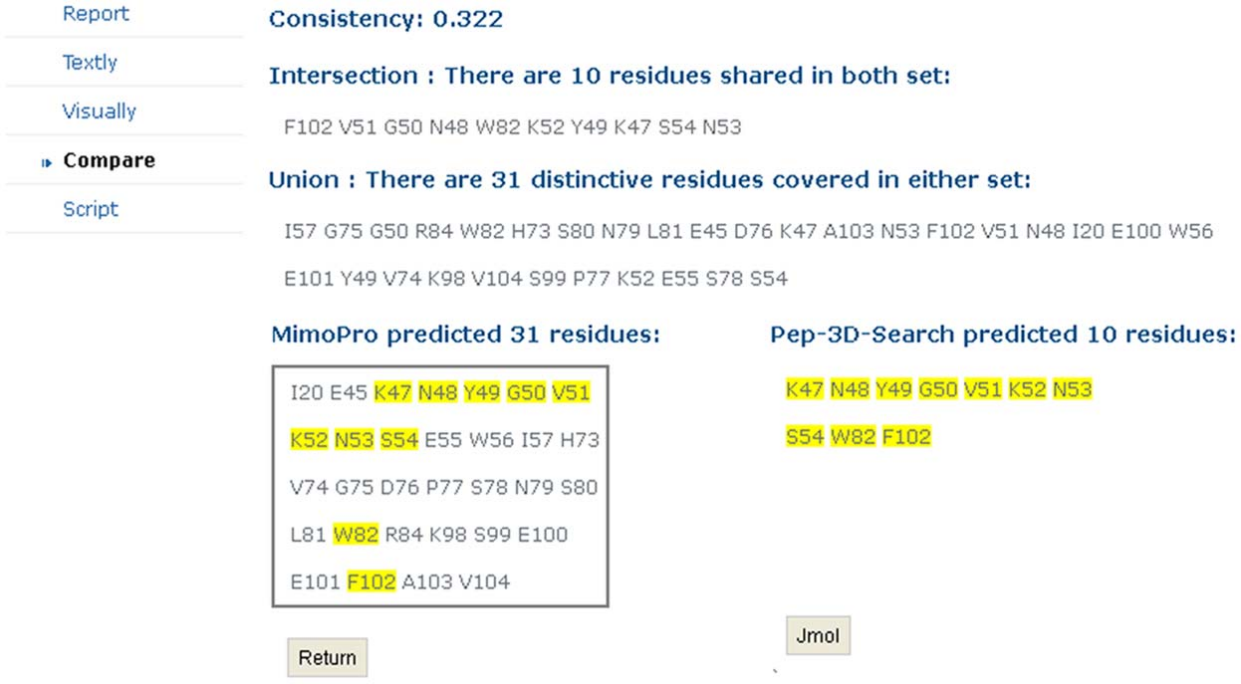
An example of text presentation of Intersection and Union of PepMapper. Results of *Intersection* and *Union* are listed in text. The overlapped candidate peptides of 1JRH are highlighted in yellow in the two boxes.

**Figure 4:**
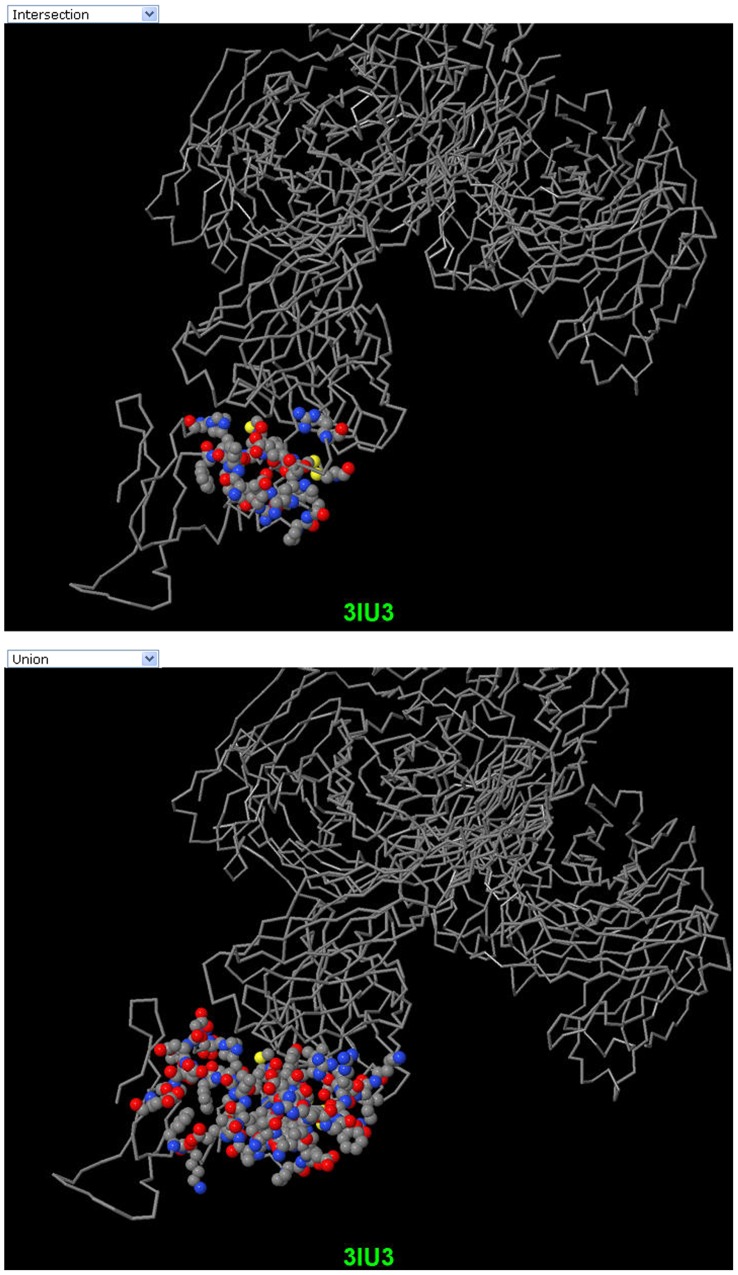
3D images of the results from Intersection and Union of PepMapper. The candidate epitopes of 3IU3 are shown in the shape of spacefill and cpk color format with the rest amino acids in backbone. The image on the top is the result from *Intersection* of PepMapper whereas the bottom one is the result from *Union* of PepMapper. It can be clearly seen on these images that *Intersection* provides more confined prediction as most of the residues lie on the interface whereas *Union* outlines a larger area that may cover (part of) the potential epitopic region.

If a user selects either MimoPro or Pep-3D-Search, PepMapper works almost exactly as either does individually, except some possible minor variations in results of this modified version of Pep-3D-Search from its original version [4]. If a user selects the Both option, PepMapper executes both MimoPro and Pep-3D-Search concurrently without mutual interference. The user will get a complete report of processed results through the emailed link. The user can access the normal result of either MimoPro or Pep-3D-Search as each works alone. To view the results of the Both option, the user has to press Compare on the left side of the result Webpage, which will produce a new Webpage showing the text results of both Intersection and Union of the two methods (Figure 3). By clicking Jmol button on this Webpage, the 3D image of the result from either Intersection (by default) or Union can be displayed (Figure 4).

The combined operations of Union and Intersection are defined as
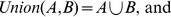
(1)

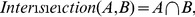
(2)where A and B are two sets of epitopic amino acids predicted by MimoPro and Pep-3D-Search respectively. Union captures the concept of exploring as many associated peptides as possible from both methods by constructing a new set that consists of not only the common epitopic amino acids in both A and B, but also all the distinctive epitopic amino acids in either A or B. Therefore, Union should be more sensitive than either method in epitope detection. Intersection realizes the concept of mutual verification from both methods by creating a new set that consists of only the common epitopic amino acids in both A and B. Hence, Intersection should be more reliable than either method in epitope detection if its outcome is positive.

Without confirmation from real experiment results, mutual verification from artificial prediction methods can only provide an indication of where the true epitopic region is likely located on the antigen surface. Ideally if both methods produce exactly the same epitopic amino acids, both Intersection and Union should return the same set of epitopic amino acids. Hence MimoPro and Pep-3D-Search share a consistency of 100% to each other on the case of epitopic prediction. On the other hand, if there is no common epitopic amino acid in the results from the two methods, MimoPro and Pep-3D-Search have no consistency to each other on the case of epitopic prediction, which implies a failure in mutual verification of epitopic prediction between the two methods. However, this failure in mutual verification only means that the two methods cannot support each other on the case under study, but it does not mean that the predicted epitopic regions by either method are not related to the genuine epitope. Other approaches are needed to verify the epitopic regions predicted by either method.

Commonly, consistency of epitope prediction from the two methods falls between 0 and 1. To present this indication numerically, we define the Consistency of the two methods in epitope prediction as
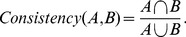
(3)


The higher the Consistency, the larger the overlapped area of predicted epitopic regions by both methods; hence it is an indirect indication that a genuine epitope is more likely to be found around the overlapped area on the antigen surface under study.

### Implementation of PepMapper Server

PepMapper has been implemented using C++ as a Web-based tool located at http://informatics.nenu.edu.cn/PepMapper. It is currently deployed on Linux using tomcat server 6.0 and has been tested using many popular Web browsers, such as IE7-9, Firefox, and Opera. Three options, MimoPro, Pep-3D-Search, and their combination, are available for the users to choose for the purpose of their applications. Note that the original Web tool of Pep-3D-Search was implemented using VB.NET. Pep-3D-Search in PepMapper is re-implemented using C++ and a modified ACO algorithm is adopted for more efficient searching (See Table S1 for details).

If a user has multiple requests and needs the results to be returned fairly quickly, it is suggested to choose MimoPro for meeting such purpose because MimoPro is arguably the fastest in processing [20]. If the user wants to verify the results, it is suggested to choose the combination mode.

When accessing PepMapper online, *Mapping* is the default interface displayed. The input to PepMapper is the structure of a chosen antigen and the peptide library screened from the corresponding antibody. The user needs to specify both the identifier of an antigen in the PDB database through its *PDB_ID* and the identifier of the interacting chain through *Chain No*. The user then needs to specify at least one peptide in the box labeled as *Mimotopes*. The peptides should be grouped in the *FASTA* format or just in separated lines of sequences. At last, the user needs to provide a valid email address in the text box. By clicking *Query*, PepMapper begins processing and the results will be sent to the user through the email provided.

The result from PepMapper is a candidate epitope along with the alignment for each peptide sequence. Users can see the result in three ways: text/table, 3D graphics, and Rasmol scripts.

In text/table format, texts are used to list all potential amino acids. The resultant alignments for individual peptide sequences are tabulated with corresponding *P*-values. In 3D graphics through Jmol, the candidate epitope is shown in filled balls and the other amino acids are shown as backbones by default (Figure 4). Results can also be presented in Rasmol script that can be downloaded by clicking the link provided. This is useful when the network connection is poor.

A new function *Compare* is also provided to make mutual verification easier between the results of the two methods. By clicking Compare on the left in the result Webpage, the peptides constituting the candidate epitope are displayed in two boxes corresponding to both methods. Clicking Compare under the left box will return a new Webpage that shows the results of both Intersection and Union from both methods (Figure 3), which can also be viewed as 3D images by clicking Jmol button on this Webpage (Figure 4).

## Results and Discussion

### Data Preparation

The task of epitope prediction based on the peptide set is to map it back to the epitopic region on an antigen that interacts with the target molecule during in vitro screening. Although there may be other epitopes on the antigen surface, we only consider the active epitope in the designated context and regard the rest part of the antigen as nonepitope.

For the test cases that correspond to the same epitope and same reference antigen structure in PDB database [18], [21] but different mimotope sets, we retain only one representative to avoid the possible bias caused by the duplication. Those cases with antigen smaller than 80 amino acids are excluded because they are too small to reflect the performance. Based on these rules, the final dataset was constructed by 27 test cases (Table 1).

**Table 1:** Test cases for validation and assessment.

PDB_ID	Target	Template	Mimotopes#
1JRH	A6, IgG1	IFNgammaR	59×5
1BJ1	rhuMAb	vascular endothelial growth factor	36×6, 3×5, 2×4
1G9M	17b	gp120	10×14, 1×12
1E6J	13B5	p24	14×14, 2×7
1N8Z	Herceptin	Her-2	5×12
1IQD	BO2C11	Coagulation factor VIII	27×12
1YY9	Cetuximab	Epidermal Growth Factor Receptor	3×10
2ADF	82D6A3, IgG	human von Willebrand factor (vWF)	2×15, 3×6
1ZTX	E16	West Nile Virus envelope glycoprotein(WNV E)	3×13, 19×14
3IU3	basiliximab	Interleukin-2 receptor subunit alpha	6×9
2GHW	80R	Spike glycoprotein	9×16, 11×15, 17×14, 4×13
3IU3	Interleukin-2 receptor subunit alpha	Anti-CD25 monoclonal antibody basiliximab	6×9
2NY7	Anti-gp120 monoclonalantibody b12	Surface protein gp120 (SU)	1×12 1×15
1AVZ	Fyn	SH3 domain Nef Bovine	8×11, 10×12
1HX1	Hsc70	Bag chaperone regulator	8×15
1SQ0	Platelet glycoprotein Ib alpha chain	von Willebrand factor (vWF)	3×11
1MQ8	ICAM-1	Integrin alpha-L beta-2	1×14
1II4	FGFR-2	HBGF-2	30×7
1WLP	NCF-1	Cytochrome b-245	30×9, 3×8
2GRX	Ferrichrome-iron receptor	Protein tonB	13×8
2GSK	von Willebrand factor (vWF)	Platelet glycoprotein Ib alpha chain	6×9
1FLT	VEGFR-1	VEGF-A	7×4
1SHY	Hepatocyte growth factor	Hepatocyte growth factor receptor	1×13, 1×12
1D4V	Tumor necrosis factor ligandsuperfamily member 10	Tumor necrosis factor receptor superfamily member 10B	13×9
1EER	Erythropoietin	Erythropoietin receptor	1×10
3EZE	Phosphocarrier protein HPr	Phosphoenolpyruvate-protein phosphotransferase	6×15

# Number of peptides × peptide length.

* 1N8Z^*^ shares the same crystal complex with 1N8Z in PDB database.

In order to analyze the performances of Pep-3D-Search, MimoPro and PepMapper, the outcome is assessed by a number of measurements, including sensitivity (Se), specificity (Sp), and precision (Pr) defined as follows:
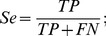
(4)

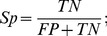
(5)

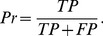
(6)In these expressions, *TP* is the number of predicted epitopic amino acids proven to be the true epitopic amino acids. *FP* is the number of predicted epitopic amino acids proven not to be the true epitopic amino acids. *TN* is the predicted non-epitopic amino acids proven not to be the true epitopic amino acids. *FN* is the number of predicted non-epitopic amino acids proven to be the true epitopic amino acids. We use *PE* to denote the number of all predicted epitopic amino acids (the sum of *TP* and *FP*).

To demonstrate the improved performance of PepMapper over either MimoPro or Pep-3D-Search alone, we first present the results from MimoPro and Pep-3D-Search run separately and then the results from the combined operations.

### Results from MimoPro and Pep-3D-Search

Table 2 presents the evaluation results from the two methods run separately. The performances of prediction from MimoPro and Pep-3D-Search varied in different test cases. MimoPro provided some good results on 2ADF_A, 3EZE_B, 1JRH_I, 1BJ1_H, 1N8Z_C and 1ZTX_E with sensitivity exceeding 0.8 and specificity higher than 0.6; meanwhile the worst results were observed in 1YY9_A, 2NY7_G, 2GRX_A, 1D4V_B, 3BT1_A and 1HX1_A with sensitivity approaching to 0. For the rest cases, the sensitivity of prediction was between 0.25 and 0.6 and the specificity is consistently higher than 0.8. Comparatively, Pep-3D-Search gave better results in 1HX1_A and 1D4V_B, in which MimoPro failed to predict any epitopic amino acids. However, Pep-3D-Search failed in 2ADF_A, 1EER_A and 1MQ8_B whereas MimoPro produced useful results. On average, MimoPro gives better results in terms of sensitivity (0.446) and precision (0.267), but slightly worse than Pep-3D-Search in specificity.

**Table 2:** Statistical results of Pep-3D-Search and MimoPro.

PDB_ID	MimoPro				Pep-3D-Search			
	TP/PE	Se	Sp	Pr	TP/PE	Se	Sp	Pr
3IU3_I	16/34	0.571	0.908	0.471	12/30	0.429	0.908	0.400
1HX1_B	14/38	0.583	0.727	0.368	5/32	0.208	0.693	0.156
1YY9_A	0/43	0.000	0.928	0.000	0/41	0.000	0.931	0.000
2ADF_A	13/24	0.867	0.937	0.542	0/31	0.000	0.822	0.000
1IQD_C	9/39	0.563	0.786	0.231	8/37	0.500	0.793	0.216
2GHW_A	14/38	0.483	0.862	0.368	8/36	0.276	0.839	0.222
2NY7_G	0/40	0.000	0.863	0.000	2/41	0.077	0.866	0.049
1WLP_B	9/47	0.310	0.651	0.191	17/45	0.586	0.743	0.378
1G9M_G	9/50	0.600	0.896	0.180	11/35	0.733	0.939	0.314
1E6J_P	11/42	1.000	0.844	0.262	11/29	1.000	0.910	0.379
2GRX_A	0/32	0.000	0.954	0.000	0/24	0.000	0.965	0.000
2GSK_A	8/40	0.190	0.942	0.200	0/32	0.000	0.942	0.000
1FLT_X	7/35	0.333	0.622	0.200	4/23	0.190	0.743	0.174
1SHY_A	6/44	0.261	0.820	0.136	7/44	0.304	0.825	0.159
1SQ0_A	8/34	0.296	0.861	0.235	7/35	0.259	0.850	0.200
1D4V_B	0/30	0.000	0.792	0.000	5/39	0.263	0.764	0.128
3BT1_A	0/40	0.000	0.672	0.000	0/27	0.000	0.779	0.000
1EER_A	7/26	0.184	0.852	0.269	0/11	0.000	0.914	0.000
1MQ8_B	7/30	0.412	0.856	0.233	0/16	0.000	0.900	0.000
3EZE_B	24/35	0.960	0.817	0.686	21/38	0.840	0.717	0.553
1II4_A	23/41	0.622	0.847	0.561	21/42	0.568	0.822	0.500
1HX1_A	0/46	0.000	0.879	0.000	6/37	0.286	0.918	0.162
1JRH_I	20/31	0.952	0.851	0.645	9/10	0.429	0.986	0.900
1BJ1_H	15/36	0.882	0.899	0.417	12/36	0.706	0.884	0.333
1N8Z_C	18/38	0.900	0.966	0.474	17/34	0.850	0.971	0.500
1ZTX_E	13/39	0.813	0.694	0.333	11/35	0.688	0.718	0.314
1AVZ_B	10/32	0.625	0.812	0.313	7/38	0.438	0.735	0.184
		0.460	0.835	0.271		0.357	0.847	0.230

TP: number of true positive; PE: number of predicted epitope; Se: sensitivity; Sp: specificity; Pr: precision.

Both MimoPro and Pep-3D-Search failed in 1YY9_A, 2GRX_A, 1EER_A, and 3EZE_B. Consequently, PepMapper failed as well. We think that this failure could be attributed to a number of factors, including the quality of the experimental data and the complexity of the predicting tasks. For instance, we found that the mimotope set used for predicting the epitopic region of 2GRX_A is screened against the whole complementary protein rather than the restricted region of the two interacting proteins. Therefore it is reasonable to suppose that there may be multiple regions on the surface of the target antigen to which the mimotope can bind. As a result, the mimotopes may bind to the regions that are different from the preferable region.

Additionally, the limited number of mimotopes (1D4V_B, 1EER_A) and surface amino acids may also complicate the matter, since the small number of mimotopes contains little information for locating the epitopic region, especially where too many surface amino acids exist. Furthermore, even though the dataset for our experiments has been the largest ever reported publicly, a few bad results can still greatly influence the statistical results.

### Results from PepMapper

The Intersection operation of PepMapper captures the idea of mutual verification of epitope prediction. Intuitively, the more the commonly shared peptides in the same area are, the more likely the area to be a part of an epitope is. On average, this operation has the highest specificity of 0.930 and a high precision of 0.256 compared to that of the Union, MimoPro, and Pep-3D-Search (Tables 2 & 3). However, its sensitivity is the lowest because some epitopic amino acids predicted by either method but not in common are left out in the calculation. This also reveals the weakness of the Intersection operation of PepMapper, i.e., in case of no overlapping between the two methods, it does not mean that no epitopic sites may be predicted by either MimoPro or Pep-3D-Search alone. 1MQ8 is such a case without common peptides, but MimoPro still predicts some positive epitopic sites.

Fortunately, the union operation of PepMapper complements the weakness of Intersection operation by joining the results from the two methods together to increase the size of potential epitopic sites. The Union operation produced the best performance in sensitivity but the worst in precision and specificity compared to that of the Intersection, MimoPro, and Pep-3D-Search (Tables 2 & 3). This is because the increased size of potential epitopic sites brought in by the Union operation also contains more false positives in the candidates.

**Table 3:** Statistical results of PepMapper.

PDB_ID	Inter*/Union	Consistency	Intersection				Union			
			TP/PE	Se	Sp	Pr	TP/PE	Se	Sp	Pr
3IU3_I	18/46	0.391	9/18	0.321	0.954	0.500	19/46	0.679	0.862	0.413
1HX1_B	18/52	0.346	4/18	0.167	0.841	0.222	15/52	0.625	0.580	0.288
1YY9_A	20/64	0.313	0/20	0.000	0.967	0.000	0/64	0.000	0.893	0.000
2ADF_A	0/42	0	0/0	0	1	0	13/55	0.867	0.759	0.236
1IQD_C	23/53	0.434	7/23	0.438	0.886	0.304	10/53	0.625	0.693	0.189
2GHW_A	20/54	0.37	8/20	0.276	0.931	0.400	14/69	0.483	0.684	0.203
2NY7_G	6/75	0.08	0/6	0.000	0.979	0.000	2/75	0.077	0.749	0.027
1WLP_B	29/63	0.46	8/29	0.276	0.807	0.276	18/63	0.621	0.587	0.286
1G9M_G	13/72	0.181	7/13	0.467	0.985	0.538	13/72	0.867	0.851	0.181
1E6J_P	28/43	0.651	11/28	1.000	0.915	0.393	11/43	1.000	0.839	0.256
2GRX_A	17/39	0.436	0/17	0.000	0.975	0.000	0/39	0.000	0.943	0.000
2GSK_A	4/68	0.059	0/4	0.000	0.993	0.000	8/68	0.190	0.891	0.118
1FLT_X	8/50	0.16	0/8	0.000	0.892	0.000	11/50	0.524	0.473	0.220
1SHY_A	29/59	0.492	5/29	0.217	0.886	0.172	8/59	0.348	0.758	0.136
1SQ0_A	27/42	0.643	7/27	0.259	0.893	0.259	8/42	0.296	0.818	0.190
1D4V_B	15/54	0.278	0/15	0.000	0.896	0.000	5/54	0.263	0.660	0.093
3BT1_A	0/67	0	0/0	0	1	0	0/67	0.000	0.451	0.000
1EER_A	1/36	0.028	0/1	0.000	0.992	0.000	7/36	0.184	0.773	0.194
1MQ8_B	0/46	0	0/0	0	1	0	7/46	0.412	0.756	0.152
3EZE_B	25/48	0.521	20/25	0.800	0.917	0.800	25/48	1.000	0.617	0.521
1II4_A	34/49	0.694	19/34	0.514	0.873	0.559	25/49	0.676	0.797	0.510
1HX1_A	0/83	0	0/0	0	1	0	6/83	0.286	0.797	0.072
1JRH_I	10/31	0.323	9/10	0.429	0.986	0.900	12/31	0.571	0.743	0.387
1BJ1_H	27/45	0.6	12/27	0.706	0.928	0.444	15/45	0.882	0.855	0.333
1N8Z_C	31/41	0.756	16/31	0.800	0.974	0.516	19/41	0.950	0.963	0.463
1ZTX_E	31/43	0.721	11/31	0.688	0.765	0.355	13/43	0.813	0.647	0.302
1AVZ_B	22/48	0.458	6/22	0.375	0.863	0.273	10/48	0.625	0.675	0.208
				0.286	0.930	0.256		0.513	0.745	0.221

* Inter stands for intersection of the results; Union stands for union of the results.

Using Consistency defined in Equation (3) as an indirect sufficient condition to judge the likelihood of successful prediction of epitope by combining both Intersection and Union, our tests tend to support its usefulness in indicating the likelihood of successful prediction (Table 3). Although the number of tests is still insufficient for us to draw any exclusive conclusion on its implication on epitope prediction, our initial analysis leads to the following indications:

- If Consistency ≥0.5, i.e., results from both MimoPro and Pep-3D-Search overlapped at least 50%, it is almost certain to find a genuine epitope around the overlapped area on the antigen surface;
- If 0.5>Consistency ≥0.25, i.e., results from both MimoPro and Pep-3D-Search overlapped between 25% and 49%, it is likely to find a genuine epitope around the overlapped area on the antigen surface;
- If 0.25>Consistency>0, i.e., results from both MimoPro and Pep-3D-Search overlapped with a portion smaller than 25%, it is still possible to find a genuine epitope around the overlapped area on the antigen surface;
- If Consistency = 0, i.e., results from both MimoPro and Pep-3D-Search not overlapped at all, PepMapper fails. Users are suggested to follow the result of either MimoPro or Pep-3D-Search or other methods for further investigation.

### Conclusion and Future Work Future Directions

PepMapper, a combination of both MimoPro and a modified version of Pep-3D-Search together, sets a collaborative Web platform, on which users can conveniently conduct peptide-epitope mappings. In addition to the normal process of either MimoPro or Pep-3D-Search alone, the combined operation of Union captures the concept of exploring as many associated peptides as possible from both methods and thus increases sensitivity in finding potential epitopic regions on a given antigen surface. The Intersection operation of PepMapper realizes largely the concept of mutual verification by the two methods and hence increases the likelihood of locating the genuine epitopic region on a given antigen with respect to the interacting peptides. The Consistency between Intersection and Union can be used as an indirect sufficient condition to assess the likelihood of successful peptide-epitope mapping.

In the future, we will consider to ensemble more methods in more rationalized ways to minimize the occurrence of nil Consistency, which should enhance the effectiveness of PepMapper in peptide-epitope mapping. Effort should also be made on refining the indication of Consistency in epitope prediction by conducting more tests for various conditions. We will try to improve the efficiency of the server through utilizing distributed and/or cloud computing as well.

#### Availability

We introduced a new server, PepMapper, to incorporate both MimoPro and Pep-3D-Search which is implemented in C++ and deployed at http://informatics.nenu.edu.cn/PepMapper. It is free for the science community and academic research. However, for commercial purposes, permission must be granted by the owner of the Web tool.

## Supporting Information

Table S1 **Adaption from former Pep-3D-Search.** To improve the time efficiency of Pep-3D-Search, we made few adaptations from the former one. These includes a quicker approach in the generating a random background distribution for scoring the best aligned paths from graph search as well as the adjustment of the key parameters. As is shown in the Table S1, the performance improved on 3IU3_I, 1D4V_B in the adapted Pep-3D-Search on which the former Pep-3D-Search failed to predict any epitopic amino acids. On average, the new Pep-3D-Search has similar sensitivity and specificity, but higher precision.
(DOC)
