# Supplementary material for: PepMapper: A Collaborative Web Tool for Mapping Epitopes from Affinity-Selected Peptides
Source: PLoS One. 2012 May 25;7(5):e37869. doi: 10.1371/journal.pone.0037869 (PMC3360666; doi:10.1371/journal.pone.0037869)
Supplement: Table S1 — Adaption from former Pep-3D-Search. To improve the time efficiency of Pep-3D-Search, we made few adaptations from the former one. These includes a quicker approach in the generating a random background distribution for scoring the best aligned paths from graph search as well as the adjustment of the key parameters. As is shown in the Table S1, the performance improved on 3IU3_I, 1D4V_B in the adapted Pep-3D-Search on which the former Pep-3D-Search failed to predict any epitopic amino acids. On average, the new Pep-3D-Search has similar sensitivity and specificity, but higher precision. (DOC) [file pone.0037869.s001.doc]

**Table S1 – Adaption from former Pep-3D-Search**

| **PDB_ID** | **Adapted** | | | | **Former** | | | |
| --- | --- | --- | --- | --- | --- | --- | --- | --- |
|  | TP/PE | Se | Sp | Pr | TP/PE | Se | Sp | Pr |
| 3IU3_I | 12/30 | 0.429 | 0.908 | 0.400 | 0/13 | 0.000 | 0.933 | 0.000 |
| 1HX1_B | 5/32 | 0.208 | 0.693 | 0.156 | 5/30 | 0.208 | 0.716 | 0.167 |
| 1YY9_A | 0/41 | 0.000 | 0.931 | 0.000 | 0/41 | 0.000 | 0.931 | 0.000 |
| 2ADF_A | 0/31 | 0.000 | 0.822 | 0.000 | 0/20 | 0.000 | 0.885 | 0.000 |
| 1IQD_C | 8/37 | 0.500 | 0.793 | 0.216 | 6/46 | 0.375 | 0.714 | 0.130 |
| 2GHW_A | 8/36 | 0.276 | 0.839 | 0.222 | 0/33 | 0.000 | 0.810 | 0.000 |
| 2NY7_G | 2/41 | 0.077 | 0.866 | 0.049 | 3/31 | 0.115 | 0.904 | 0.097 |
| 1WLP_B | 17/45 | 0.586 | 0.743 | 0.378 | 12/43 | 0.414 | 0.716 | 0.279 |
| 1G9M_G | 11/35 | 0.733 | 0.939 | 0.314 | 2/45 | 0.133 | 0.891 | 0.044 |
| 1E6J_P | 11/31 | 1.000 | 0.899 | 0.355 | 11/40 | 1.000 | 0.854 | 0.275 |
| 2GRX_A | 0/24 | 0.000 | 0.965 | 0.000 | 3/42 | 0.083 | 0.943 | 0.071 |
| 2GSK_A | 0/32 | 0.000 | 0.942 | 0.000 | 0/35 | 0.000 | 0.936 | 0.000 |
| 1FLT_X | 4/23 | 0.190 | 0.743 | 0.174 | 5/22 | 0.238 | 0.770 | 0.227 |
| 1SHY_A | 7/44 | 0.304 | 0.825 | 0.159 | 3/34 | 0.130 | 0.853 | 0.088 |
| 1SQ0_A | 7/35 | 0.259 | 0.850 | 0.200 | 6/26 | 0.222 | 0.893 | 0.231 |
| 1D4V_B | 5/39 | 0.263 | 0.764 | 0.128 | 0/34 | 0.000 | 0.764 | 0.000 |
| 3BT1_A | 0/27 | 0.000 | 0.779 | 0.000 | 16/27 | 1.231 | 0.910 | 0.593 |
| 1EER_A | 0/11 | 0.000 | 0.914 | 0.000 | 3/17 | 0.079 | 0.891 | 0.176 |
| 1MQ8_B | 0/16 | 0.000 | 0.900 | 0.000 | 2/29 | 0.118 | 0.831 | 0.069 |
| 3EZE_B | 21/38 | 0.840 | 0.717 | 0.553 | 19/26 | 0.760 | 0.883 | 0.731 |
| 1II4_A | 21/42 | 0.568 | 0.822 | 0.500 | 23/44 | 0.622 | 0.822 | 0.523 |
| 1HX1_A | 6/37 | 0.286 | 0.918 | 0.162 | 7/45 | 0.333 | 0.900 | 0.156 |
| 1JRH_I | 9/10 | 0.429 | 0.986 | 0.900 | 17/43 | 0.810 | 0.649 | 0.395 |
| 1BJ1_H | 12/36 | 0.706 | 0.884 | 0.333 | 11/36 | 0.647 | 0.879 | 0.306 |
| 1N8Z_C | 17/34 | 0.850 | 0.971 | 0.500 | 20/37 | 1.000 | 0.971 | 0.541 |
| 1ZTX_E | 11/35 | 0.688 | 0.718 | 0.314 | 6/34 | 0.375 | 0.671 | 0.176 |
| 1AVZ_B | 7/38 | 0.438 | 0.735 | 0.184 | 10/31 | 0.625 | 0.821 | 0.323 |
|  |  | 0.357 | 0.847 | 0.230 |  | 0.353 | 0.842 | 0.207 |

To improve the time efficiency of Pep-3D-Search, we made few adaptations from the former one. These includes a quicker approach in the generating a random background distribution for scoring the best aligned paths from graph search as well as the adjustment of the key parameters. As is shown in the Table S1, the performance improved on 3IU3_I,1D4V_B in the adapted Pep-3D-Search on which the former Pep-3D-Search failed to predict any epitopic amino acids. On average, the new Pep-3D-Search has similar sensitivity and specificity, but higher precision.
